# Homogeneity of ball milled ceramic powders: Effect of jar shape and milling conditions

**DOI:** 10.1016/j.dib.2016.11.070

**Published:** 2016-11-24

**Authors:** M. Broseghini, M. D’Incau, L. Gelisio, N.M. Pugno, P. Scardi

**Affiliations:** a Department of Civil, Environmental & Mechanical Engineering, University of Trento, via Mesiano, 77, 38123 Trento, Italy; b Department of Civil, Environmental & Mechanical Engineering, Laboratory of Bio-Inspired and Graphene Nanomechanics, University of Trento, via Mesiano, 77, 38123 Trento, Italy; c Center for Materials and Microsystems, Fondazione Bruno Kessler, Via Sommarive 18, 38123 Povo (Trento), Italy; d School of Engineering and Materials Science, Queen Mary University of London, Mile End Road, London E1 4NS, United Kingdom

## Abstract

This paper contains data and supporting information of and complementary to the research article entitled “*Effect of jar shape on high-energy planetary ball milling efficiency: simulations and experiments*” (Broseghini et al.,) [1]. Calcium fluoride (CaF_2_) was ground using two jars of different shape (cylindrical and half-moon) installed on a planetary ball-mill, exploring different operating conditions (jar-to-plate angular velocity ratio and milling time). Scanning Electron Microscopy (SEM) images and X-Ray Powder Diffraction data (XRPD) were collected to assess the effect of milling conditions on the end-product crystallite size. Due to the inhomogeneity of the end product, the Whole Powder Pattern Model (WPPM, (Scardi, 2008) [2]) analysis of XRPD data required the hypothesis of a bimodal distribution of sizes – respectively ground (fine fraction) and less-to-not ground (coarse fraction) – confirmed by SEM images and suggested by the previous literature (Abdellatief et al., 2013) [3,4]. Predominance of fine fraction clearly indicates optimal milling conditions.

**Specifications Table**

**Table:** 

Subject area	*Physics, Chemistry*
More specific subject area	*Materials Science, Powder technology*
Type of data	*Images, graphs, raw and analyzed data*
How data was acquired	*SEM: ESEM FEI XL30 XRPD: Rigaku PMG/VH diffractometer*
Data format	*Raw, analyzed*
Experimental factors	*Data collected on milled sample without any pre-treatment. Non-destructive tests.*
Experimental features	*SEM micrographs: standard Secondary Electron detector*
	*XRPD data: CuKα radiation, scintillation counter detector and bent graphite analyzer crystal. Slits: DS 1°, Soller 2° slits, SS 1°, secondary Soller 2°, RS (0.15 mm). Instrumental profile measured using NIST SRM 660a (LaB6 powder)*
Data source location	*Trento (via Mesiano, 77), Italy, 46°04׳N 11°07׳E*
Data accessibility	*All data are with this article*

**Value of the data**

• The planetary ball-milling process is ubiquitous in the production of nanostructured materials and modification of their properties. The choice of optimal operating conditions defines end product characteristics. Data reported in this manuscript guide the understanding of the effect of two milling parameters (jar-to-plate angular velocity ratio and milling time) on the dimensional characteristics of the end product.
• The assessment of the milling behavior (*e.g.* coexistence of fine and coarse fractions and their distributions) of a new jar design and the comparison with the standard cylindrical one can be drawn from reported data and clearly show the importance of the vial shape on the end product properties and on comminution efficiency.
• XRPD raw data could be modeled with different approaches and/or used to extract more information on *e.g.* powder homogeneity and defects content introduced by the severe deformation (see [1], [6]).

## 1 Data

**Fig. 1:**
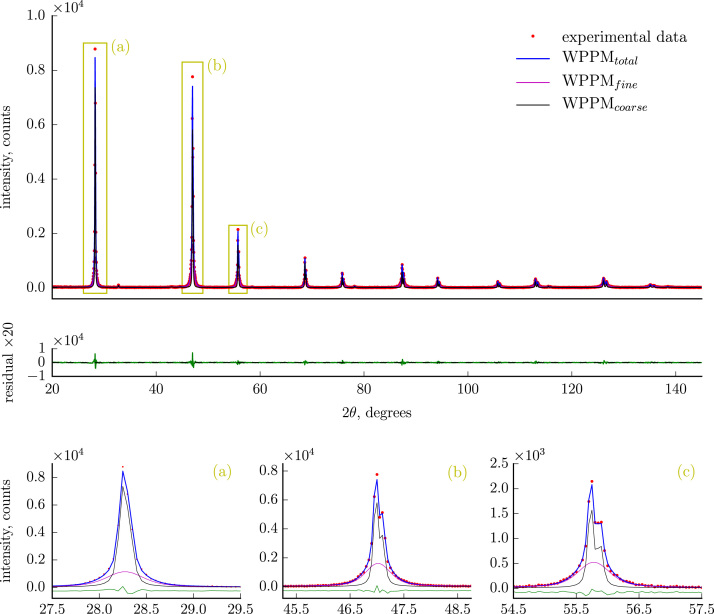
XRPD pattern of CaF_2_ ball-milled in the Half-Moon (HM) jar for 32 h and with jar (*ω*) to plate (*Ω*) velocities ratio of *ω*/*Ω*+1=−3.8 (data from Supplementary 1). Experimental data (red circles) are shown together with the WPPM profile (blue line) and their difference (residual, green line below). The contribution of a coarse-grained (black line) and a fine-grained (purple line) fluorite fraction to the model is also accentuated in the insets (a, b, c).

**Fig. 2:**
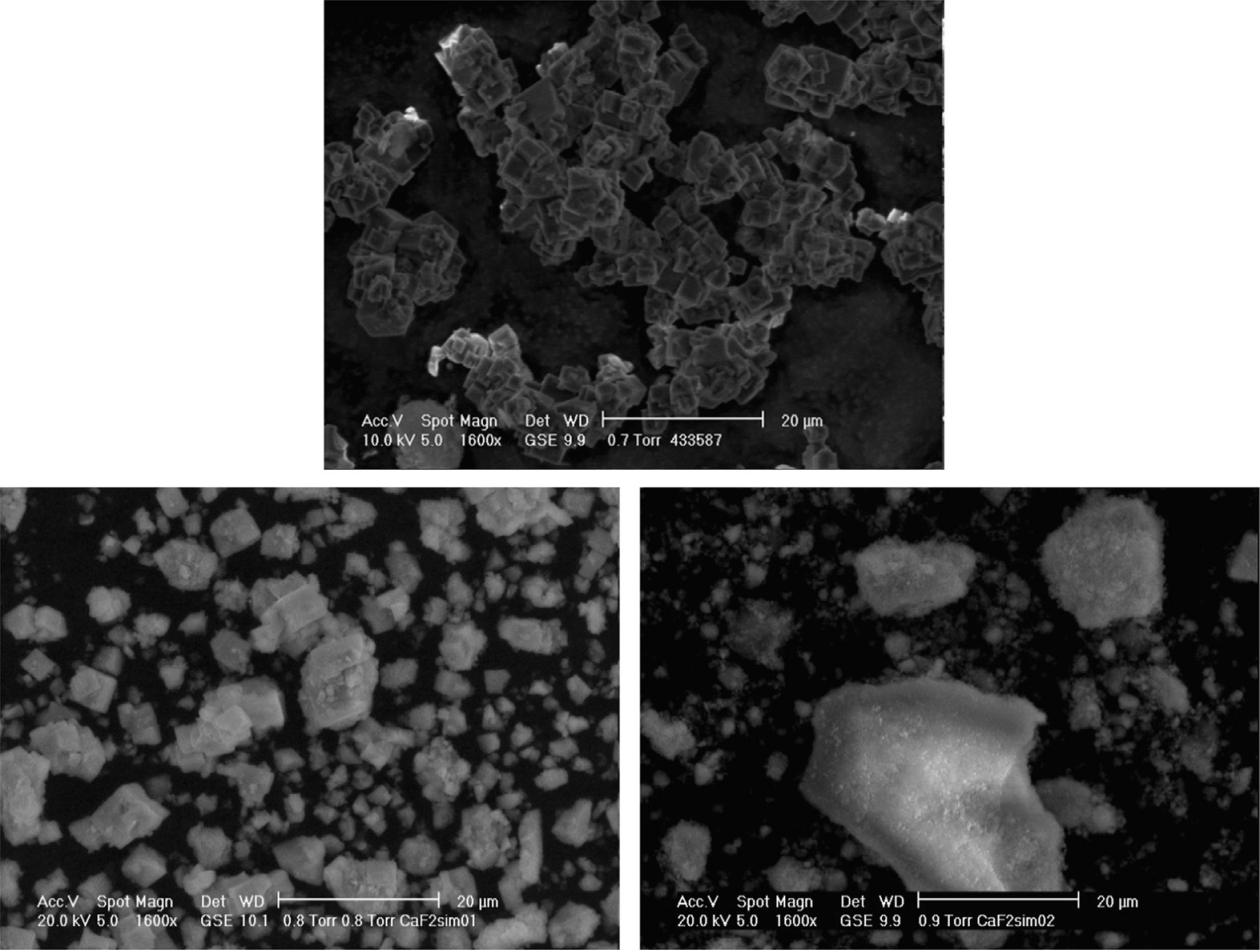
SEM micrographs of pristine powder (top) ball-milled with the cylindrical (CY, bottom left) and half-moon (HM, bottom right) jars for 8 h and with velocity ratio of *ω*/*Ω*+1=−1.0. Cubic particles typical of the pristine material are still clearly recognizable in the end product of the CY jar while almost absent after milling with the HM vial, demonstrating (and according to XRPD data reported in Fig. 3) the enhanced comminution efficiency of the HM jar under this operating condition.

**Fig. 3:**
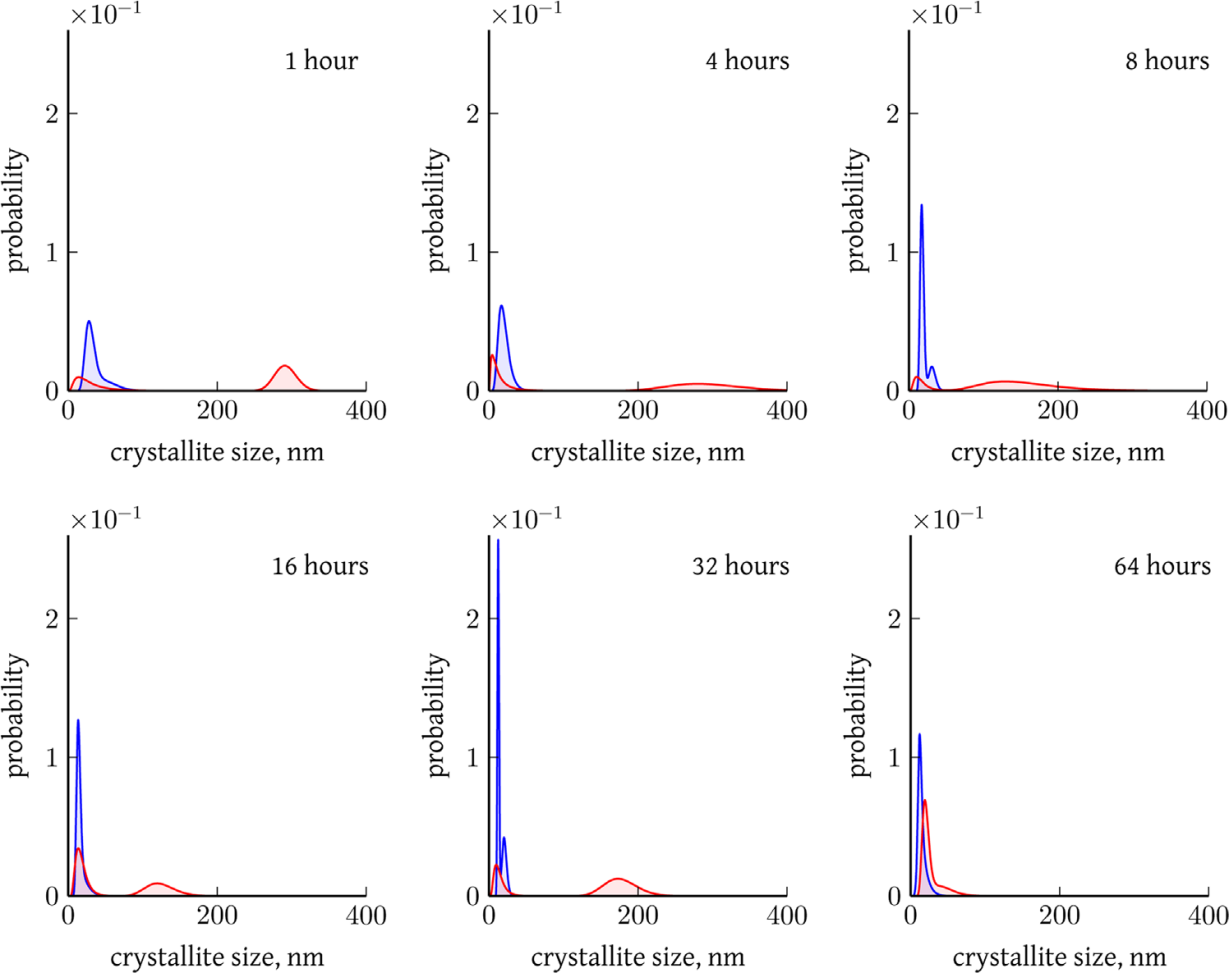
Crystallite domain size probability distribution obtained from WPPM analysis of CaF_2_ ground with the CY (red) and HM (blue) jars for increasing milling time and at *ω*/*Ω*+1=−1.0 (a complementary analysis on the effect of jar-to-plate velocity ratio is reported in Fig. 8 in [1]). Two lognormal size distributions, representing respectively the finer and the coarser (less or totally not ground crystals) fractions of the end product, were required to properly model XRPD data. The powder homogeneity increases with milling time (see also [4], [5]) and strongly depends on the vial shape, the HM jar being more effective and faster in producing a more uniform sample.

Fig. 1 illustrates a representative case of optimal modeling of XRPD data (Supplementary 1 and 2) by a WPPM method, which requires two lognormal distributions of crystallite domain sizes describing respectively a finely ground and a coarse fraction. The validity of this hypothesis, already suggested by [3,4], is further demonstrated by SEM pictures (selected cases reported in Fig. 2), clearly showing the coexistence of grains characterized by considerably different sizes. Fig. 3 compares end products size distribution obtained by WPPM analysis of data from samples milled with the cylindrical and half-moon jars for different milling times.

Supplementary 1 reports raw and analyzed XRPD data for different jar-to-plate velocities (reported for a representative case in Fig. 1; results of data analysis reported in Fig. 8 in [1]).

Supplementary 2 reports raw and analyzed XRPD data for different milling times (results of data analysis reported in Fig. 3).

## 2 Experimental design, materials and methods

### 2.1 Materials

Calcium fluoride (CaF_2_) from CARLO ERBA Reagents S.r.l.

### 2.2 Milling

Samples were milled in a Fritsch Pulverisette 4 (P4) planetary ball-mill under different operative conditions (jar-to-plate angular velocity ratio and milling time). Twelve balls were inserted in a cylindrical and in a half-moon jar (physical and geometrical properties reported in [1], [6]), designed by the authors and produced at the University of Trento (Italy).

### 2.3 Data acquisition

An ESEM FEI XL 30 was employed to acquire SEM images while XRPD data were collected using a Rigaku PMG/VH diffractometer according to the procedure reported in [1], [5].

### 2.4 XRPD data analysis

WPPM analyses [2] were performed using the software PM2K [7] and details are reported in [1].
