# Supplementary material for: Homogeneity of ball milled ceramic powders: Effect of jar shape and milling conditions
Source: Data Brief. 2016 Nov 24;10:186–91. doi: 10.1016/j.dib.2016.11.070 (PMC5149052; doi:10.1016/j.dib.2016.11.070)
Supplement: Supplementary file 1 — Supplementary material [file mmc1.pdf]

Conflict of interest

The authors declare that there is no conflict of interest involved with the manuscript “Homogeneity of ball milled ceramic powders: effect of jar shape and milling conditions”.
